# Supplementary material for: Contiguous and accurate de novo assembly of metazoan genomes with modest long read coverage
Source: Nucleic Acids Res. 2016 Jul 25;44(19):e147. doi: 10.1093/nar/gkw654 (PMC5100563; doi:10.1093/nar/gkw654)
Supplement: SUPPLEMENTARY DATA [file supp_44_19_e147__index.html]

Contiguous and accurate de novo assembly of metazoan genomes with modest long read coverage — SUPPLEMENTARY DATA 

# Contiguous and accurate *de novo* assembly of metazoan genomes with modest long read coverage

## SUPPLEMENTARY DATA

- SUPPLEMENTARY DATA
- SUPPLEMENTARY DATA
- SUPPLEMENTARY DATA
