# Supplementary material for: Contiguous and accurate de novo assembly of metazoan genomes with modest long read coverage
Source: Nucleic Acids Res. 2016 Jul 25;44(19):e147. doi: 10.1093/nar/gkw654 (PMC5100563; doi:10.1093/nar/gkw654)
Supplement: SUPPLEMENTARY DATA [file supp_gkw654_nar-03607-met-k-2015-File008.pdf]

Supplementary tables:

Supplementary Table 1: This table shows assembly statistics from all assemblies presented in this paper. All coverage and NG50 calculations assume a genome size of 130Mb. The NG50 values here are the same as the NG50s presented in Figure 5.

Supplementary Table 2: This table provides the sequence coordinates of the junctions from an assembly merging. It also lists the source of errors in 3R merged assembly chromosome arm.

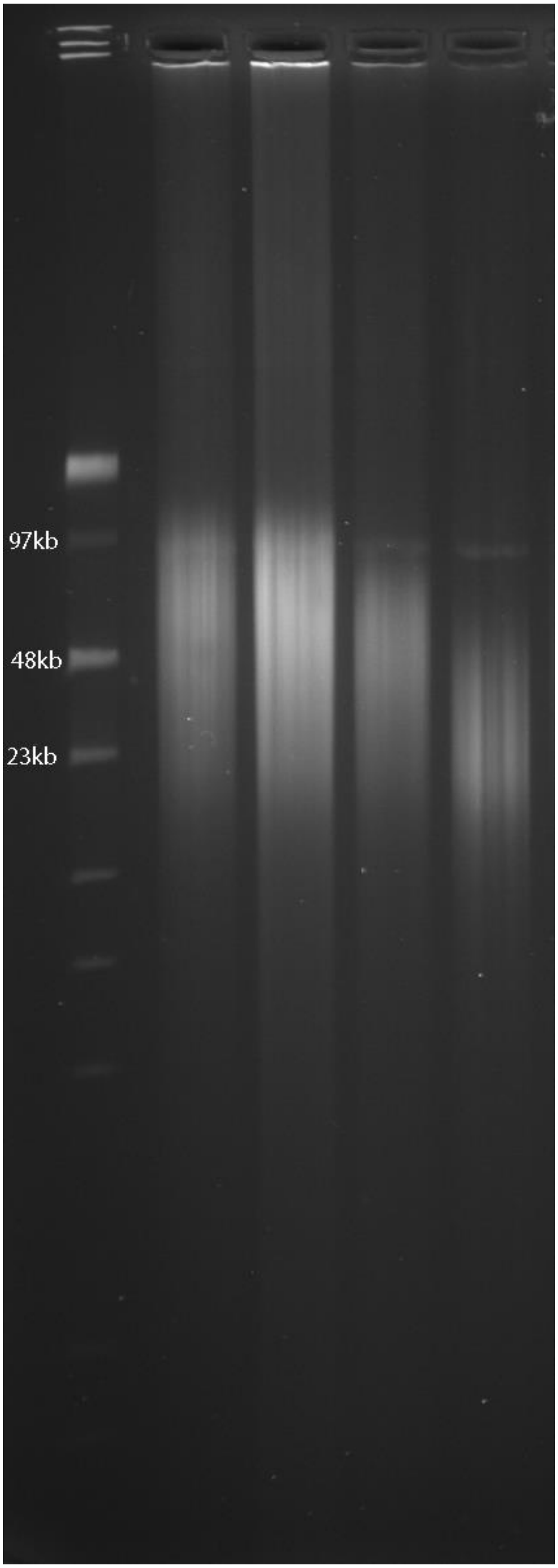

Supplementary Figure 1. A FIGE gel showing the size distribution of sheared genomic DNA fragments generated using different sized needles. From left to right: lane 1 – ladder, lane 2 – DNA sheared with 21 gauge needle, lane 3 – DNA sheared with 22 gauge needle, lane 4 – DNA sheared with 23 gauge needle, lane 5 – DNA sheared with 24 gauge needle.

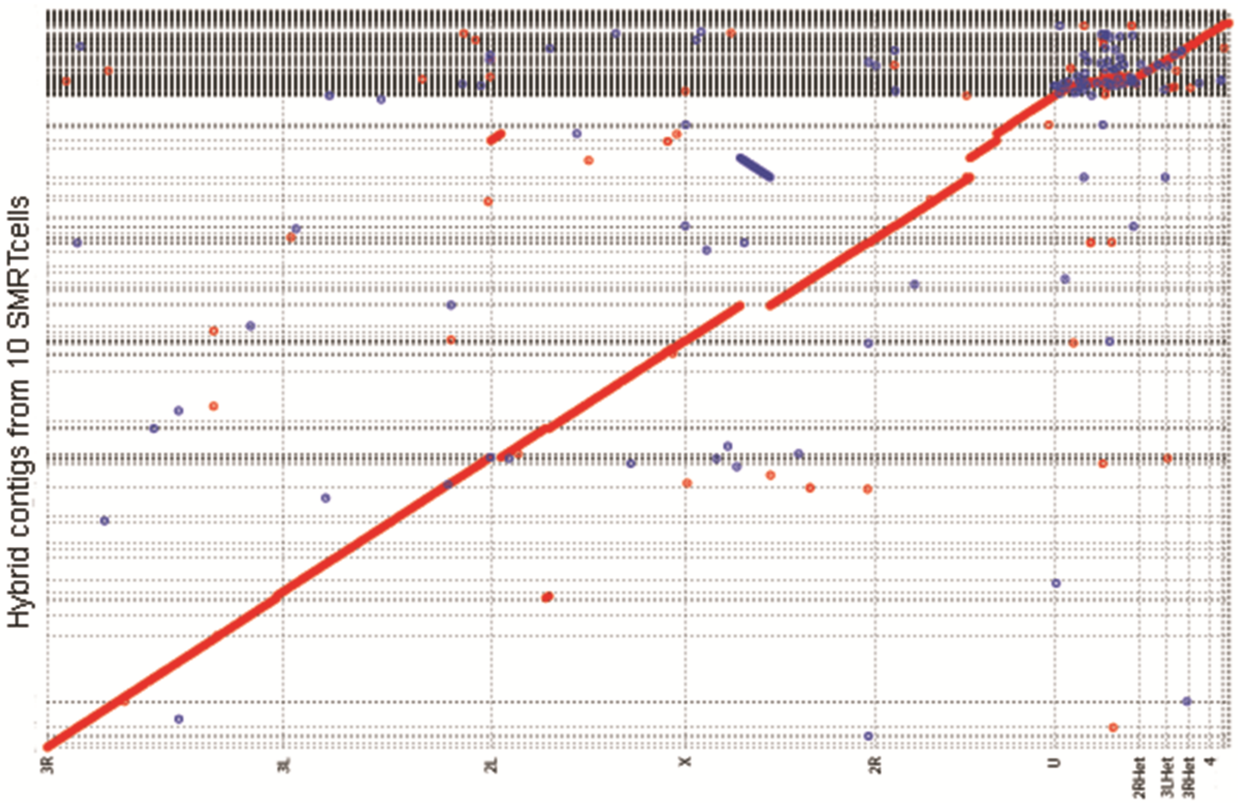

Supplementary Figure 2. An alignment plot between the 10 SMRT cell hybrid assembly and the *D. melanogaster* reference genome version 5. Red lines indicate correctly oriented contigs, while blue lines indicate inversions. A single major inversion/translocation is visible on the X chromosome. This plot was generated using MUMmer version 3.23 using the 'fat' and 'filter' plotting options.

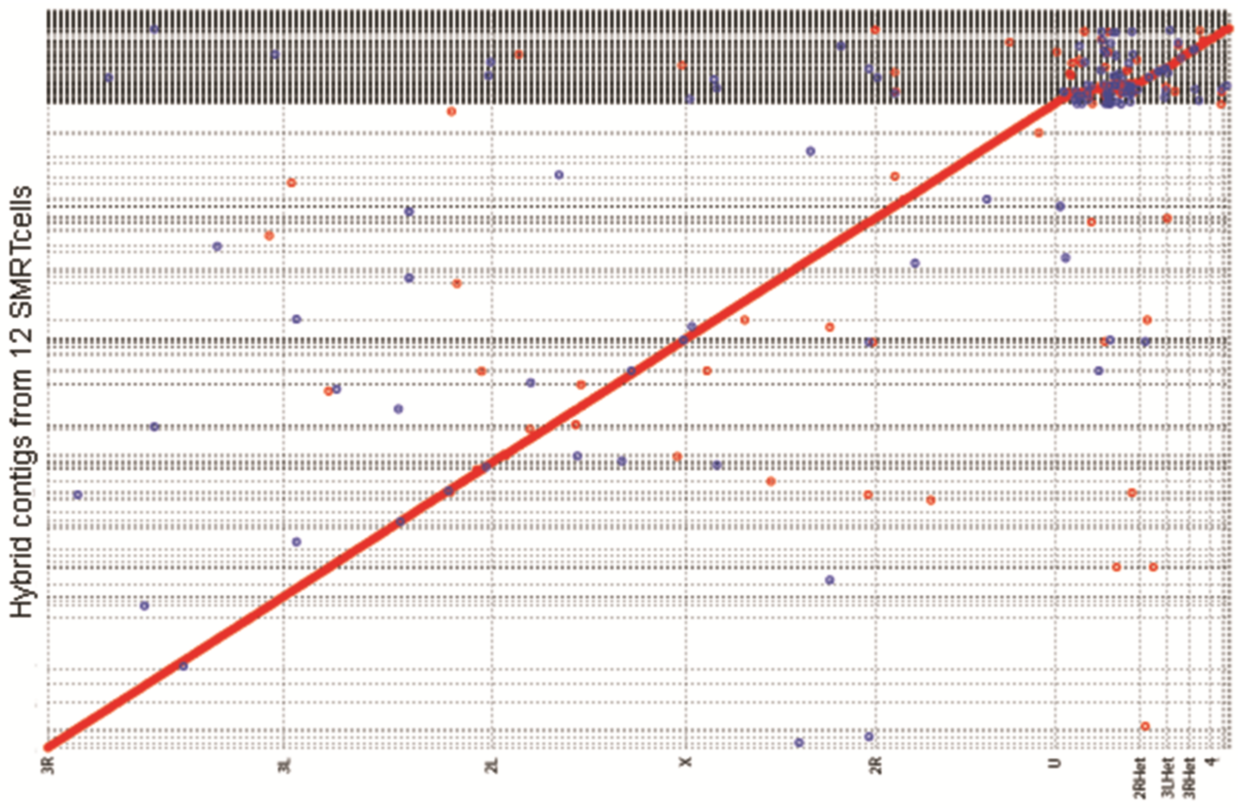

Supplementary Figure 3. An alignment plot between the 12 SMRT cell hybrid assembly and the *D. melanogaster* reference genome version 5. The inversion visible in the 10 SMRT cell hybrid assembly (Sup. Fig. 2) is not present here.

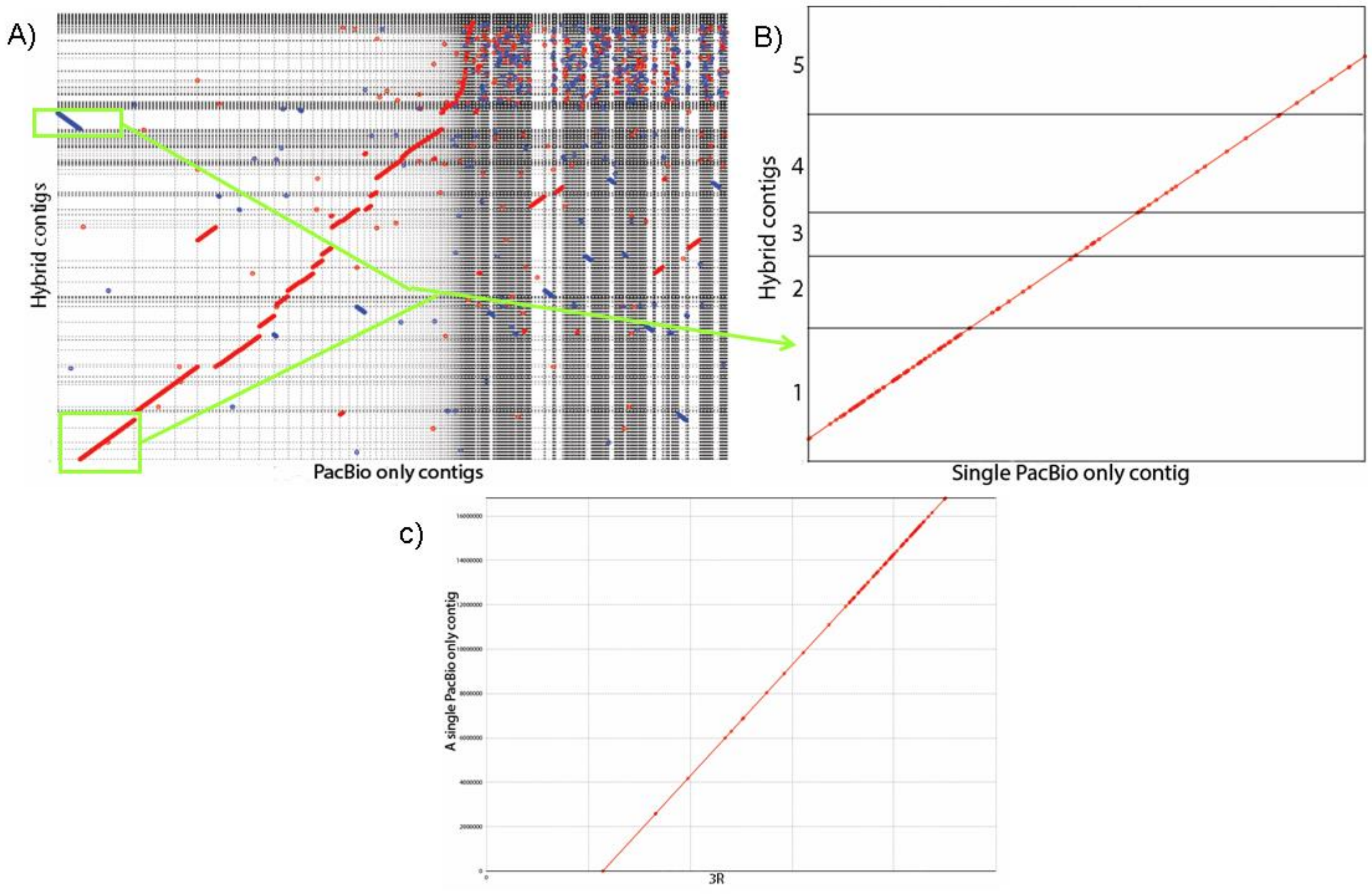

Supplementary Figure 4. a) A mummer plot depicting the alignment of the 20 SMRT cell PacBio only assembly of *D. melanogaster* to the hybrid assembly of the 50% longest reads from the same data. This plot demonstrates that large contigs produced by the PacBio only assembly are not necessarily contiguous in the complementary hybrid assembly, and vice versa. This indicates that a meta-assembly of the hybrid and PacBio only assemblies should produce a higher NG50 than either individual assembly. (b) is a zoomed in portion of (a) illustrating a PacBio only contig that contains five hybrid contigs. (c) The PacBio only contig in (b) aligns to the reference chromosome 3R.

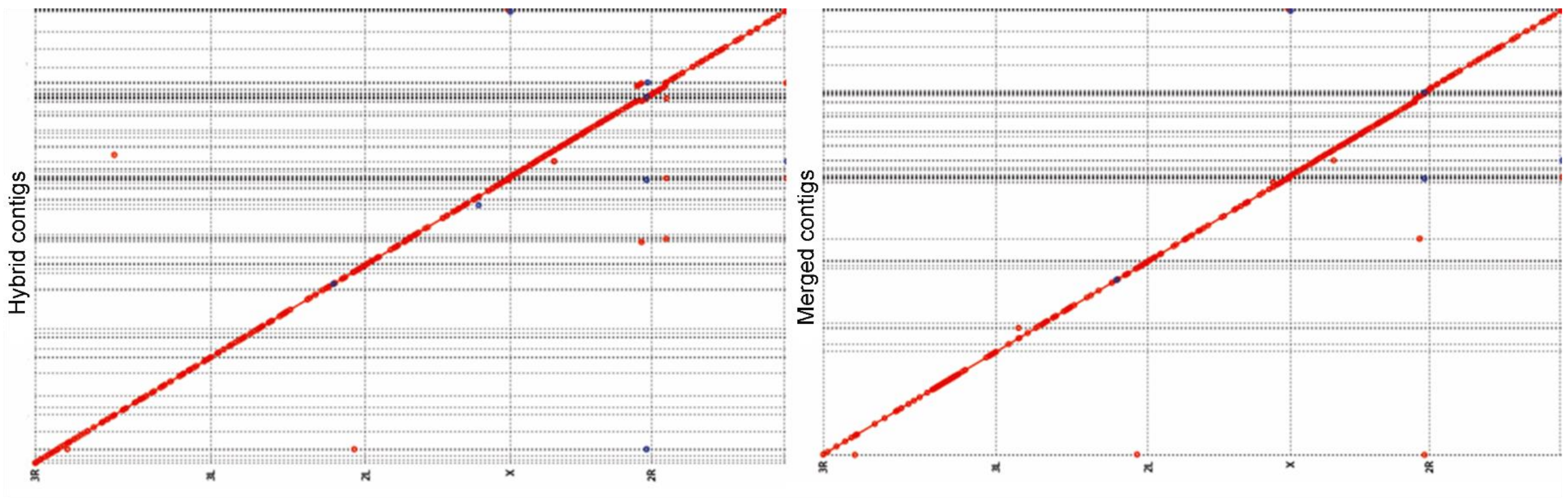

Supplementary Figure 5. A side by side comparison of the alignment of a hybrid assembly to the reference versus a merged assembly to the reference. These assemblies are both produced using the same 20 SMRTcells of data, and conform to each other closely. It is evident that the merged assembly has increased contiguity without increased misassembly.

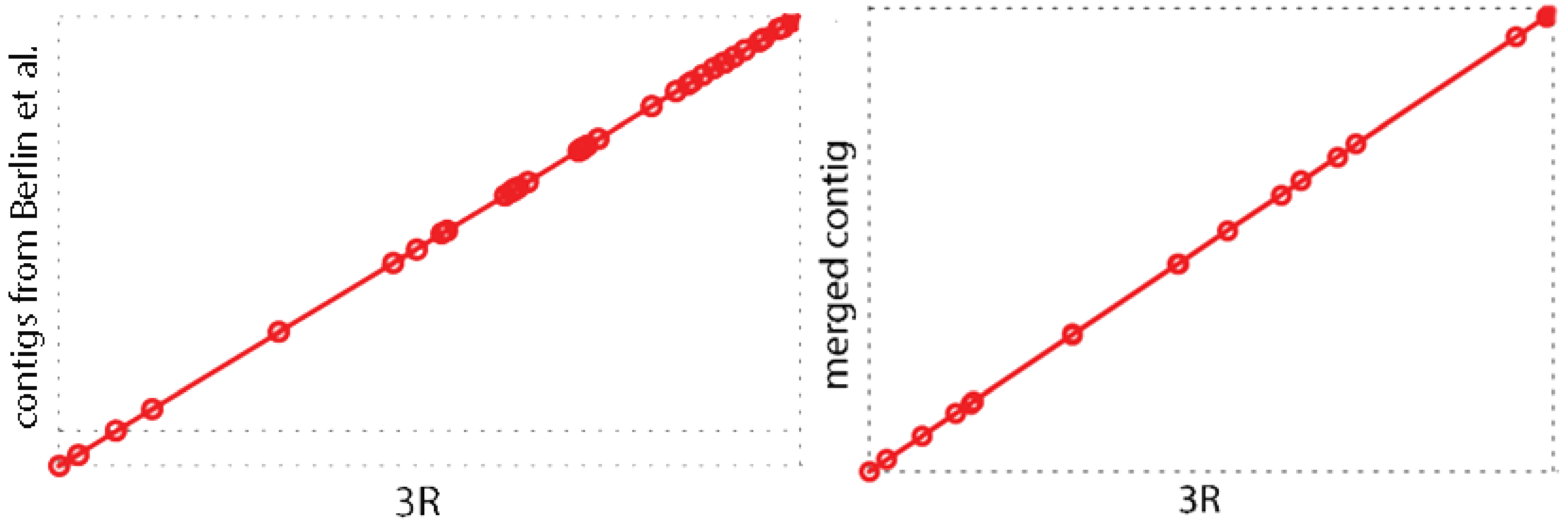

Supplementary Figure 6. A comparison of a portion of the assembly from Berlin *et al*.[1] (121X PacBio reads) to our merged assembly (52X PacBio reads), demonstrating increased contiguity when merging hybrid and PacBio only assemblies as compared to PacBio only alone.

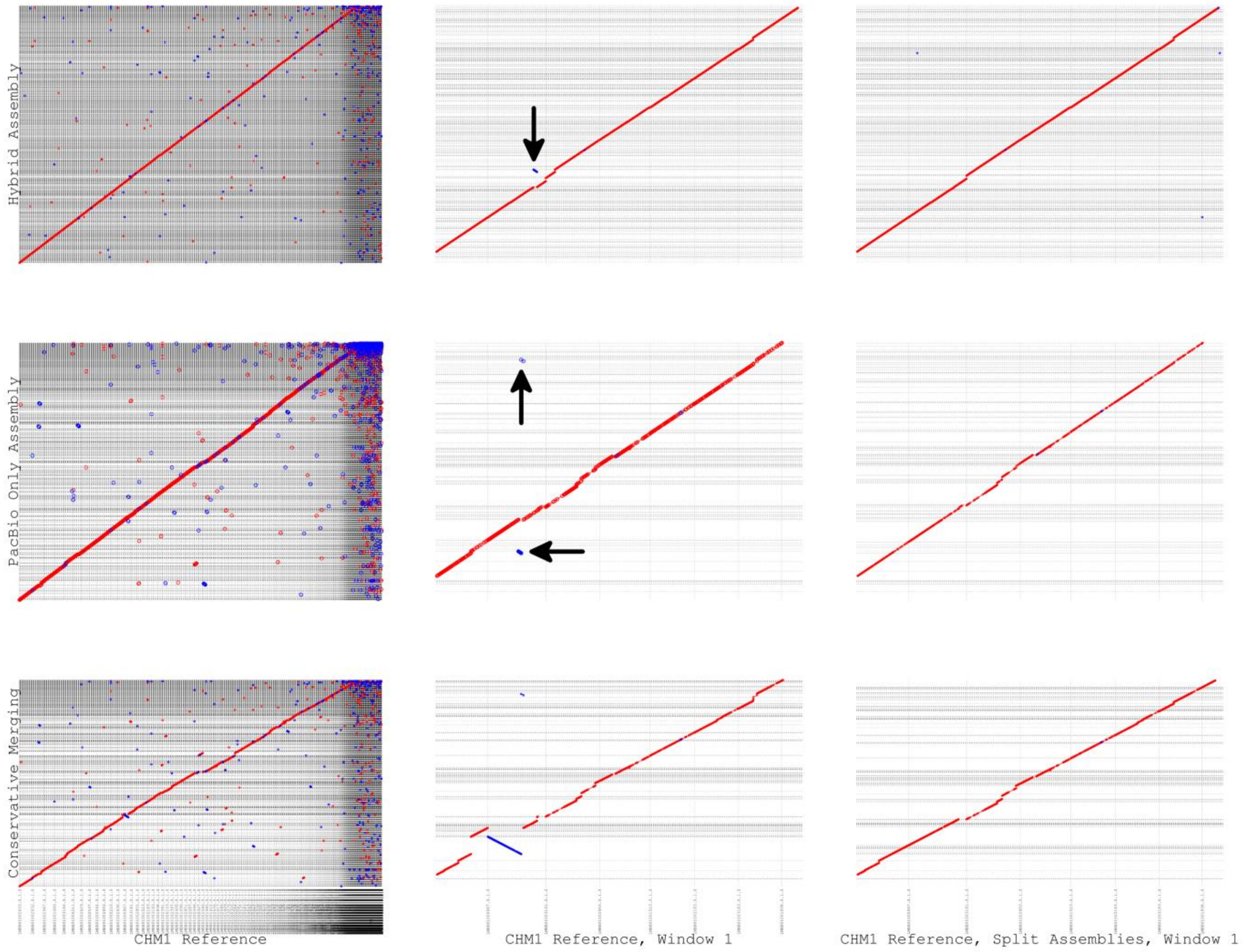

Supplementary Figure 7. Mummer dotplots between the human hybrid assembly (top row), PacBio only assembly (second row from the top), and merged assembly (bottom row) versus an extremely contiguous human genome assembly from NCBI (GenBank assembly accession GCA_001420765.1). The reference contigs are on the X-axis and the contigs from the assemblies reported here are on the Y-axis. From left to right: the first column represents the dotplots of the entire

assemblies. The second column shows a magnified view of the dotplot where the most conspicuous misassembly is present in the merged assembly and the same regions in the PacBio and hybrid assemblies. The third column shows the same view as the second column after deliberately splitting contigs (indicated by the arrows) in the component assemblies that contained inversions. The contigs were split at the inversion breakpoints (at positions 2618934 and 2099497 in the PacBio only contigs utg7180000013520 and utg7180000000047, respectively, and position 3292143 in the hybrid contig Backbone_94), and the component assemblies were merged, producing a final merged assembly that did not contain the inversions present in column two. This demonstrates that at least this set of misassemblies in the merged assembly was due to misaasemblies in the hybrid and PacBio assemblies and was not introduced by the merging process. The merged assembly was produced using quickmerge parameters hco =15, c=5, l= 5000000.

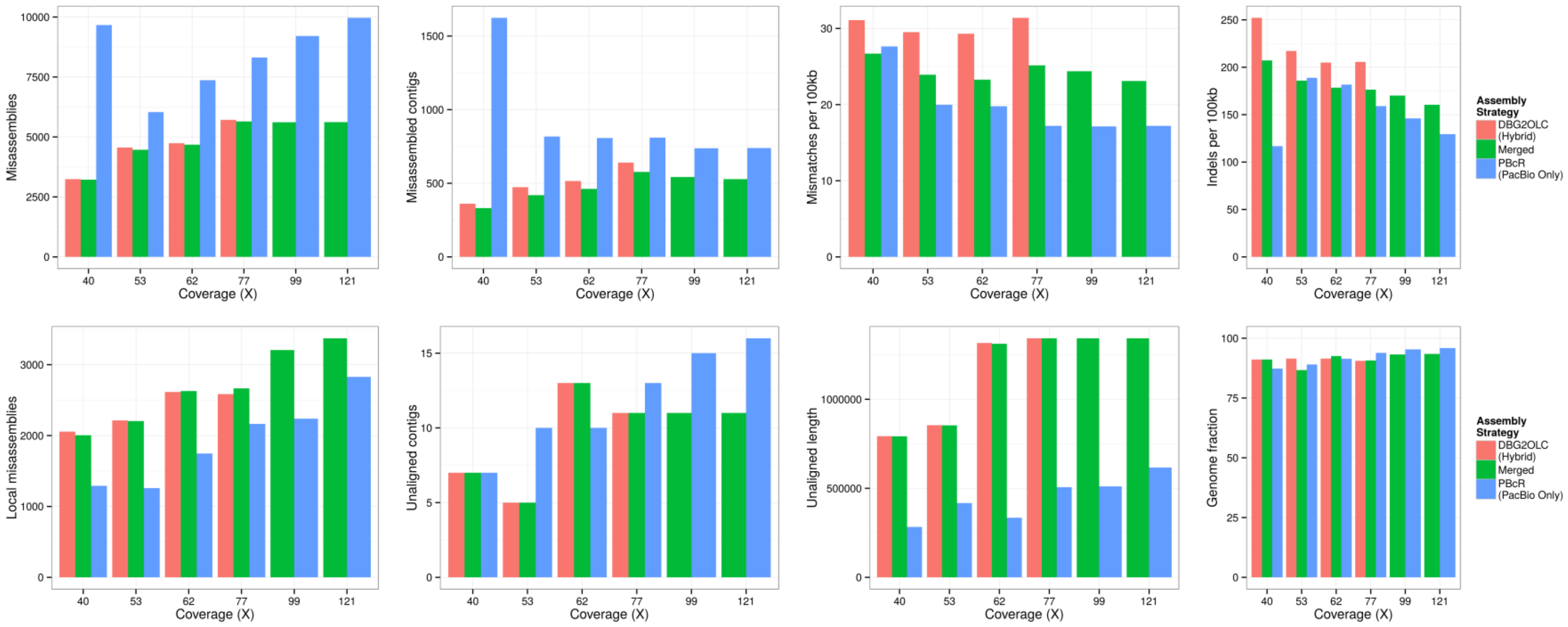

Supplementary Figure 8. Summary of unpolished assembly quality metrics from quast. All hybrid and PacBio only assemblies are based on non-downsampled reads. The merged assemblies based on up to 77X data are generated by merging the hybrid and the PacBio only assemblies made using the same amount of PacBio reads. For PacBio only assemblies made with PacBio reads >77X coverage, the hybrid assembly based on 77X PacBio reads were used for assembly merging.

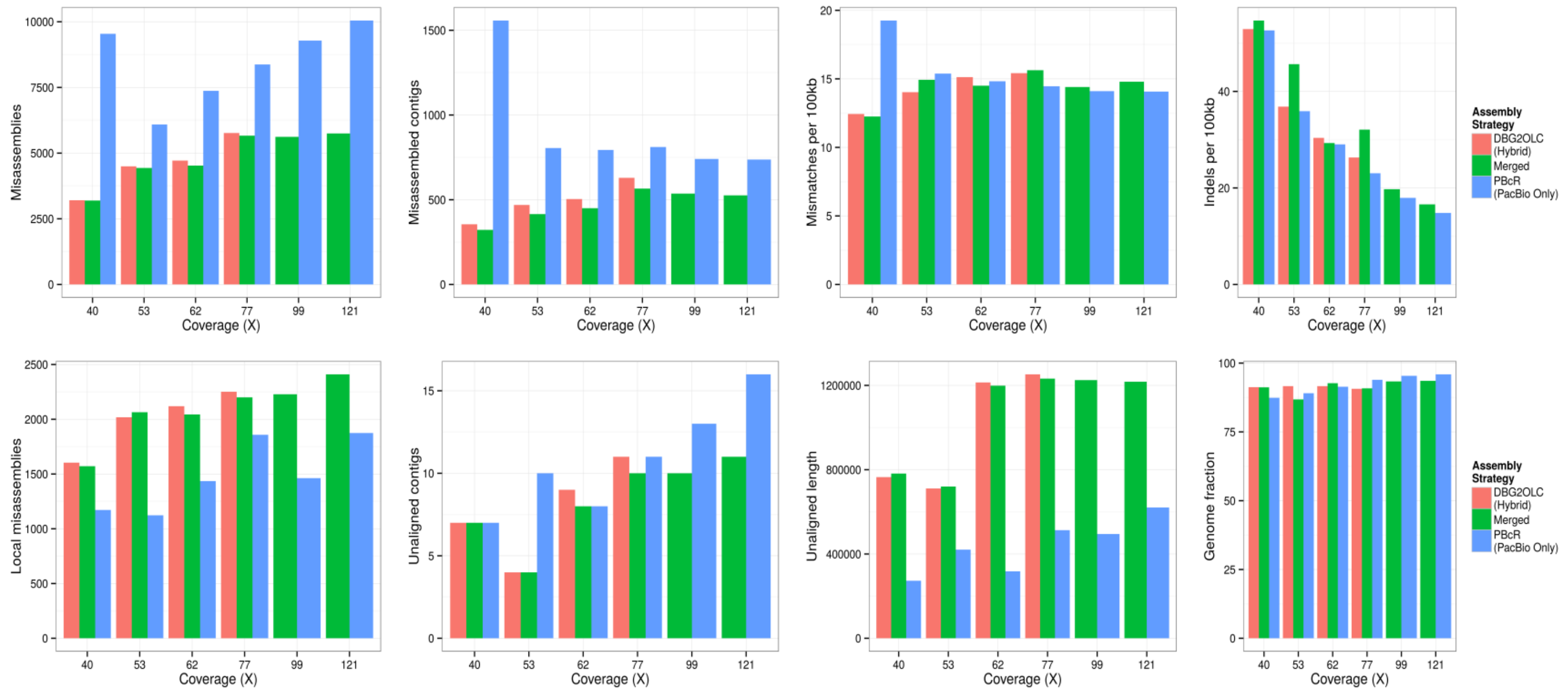

Supplementary Figure 9. Summary of quiver-polished assembly quality metrics from quast. All assemblies are same as in Supplementary Fig. 8. As evidenced here, polishing by quiver improved all assemblies.

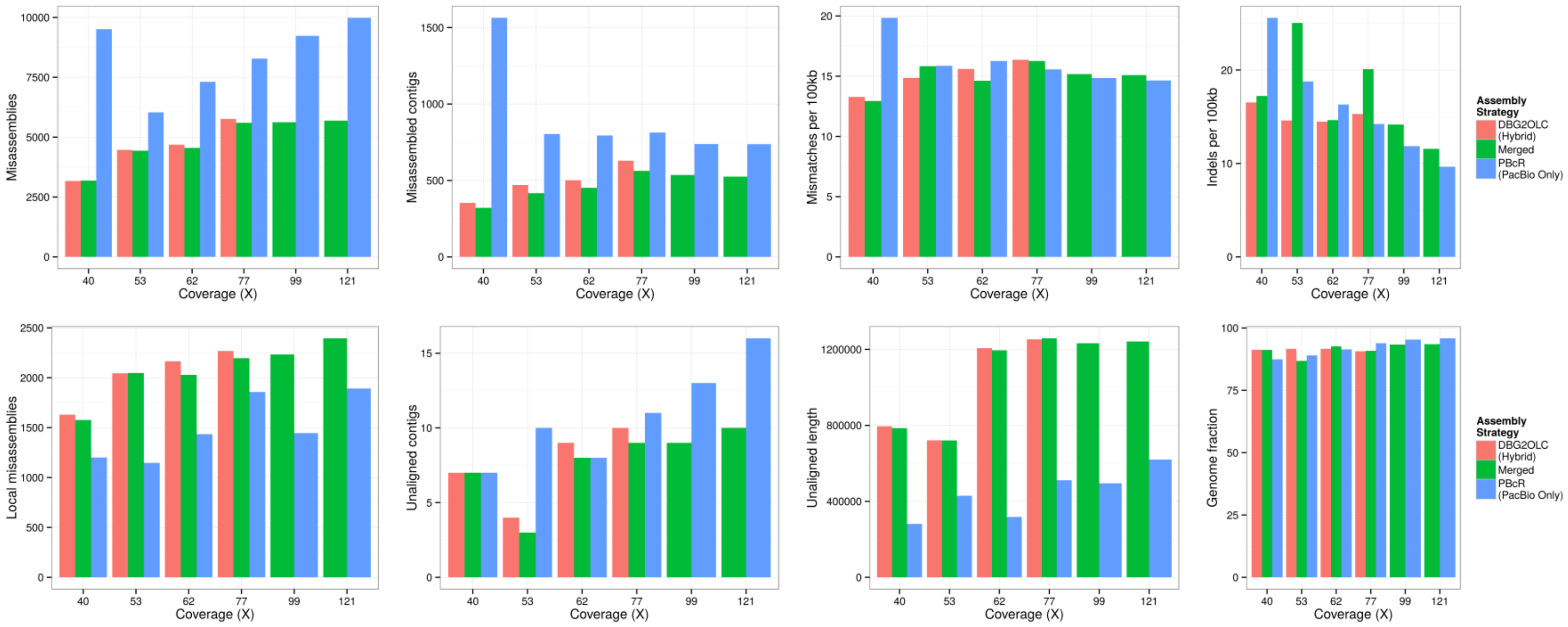

Supplementary Figure 10. Following Supplementary Fig. 9 quality metrics after both quiver and pilon polishing.

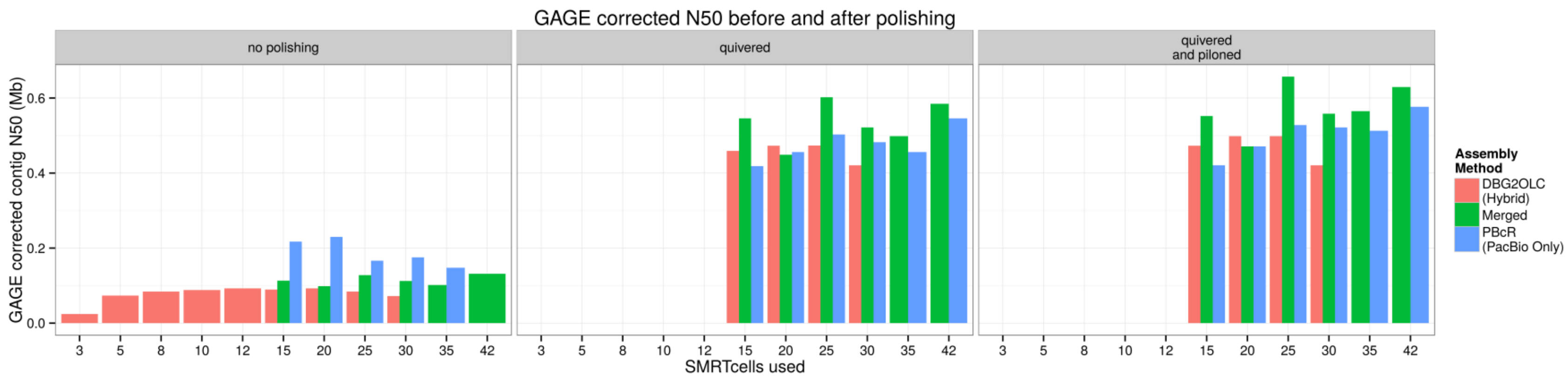

Suppelmentary Figure 11. Summary of GAGE adjusted N50 before polishing, and with polishing either by Quiver only or by both Quiver and Pilon.

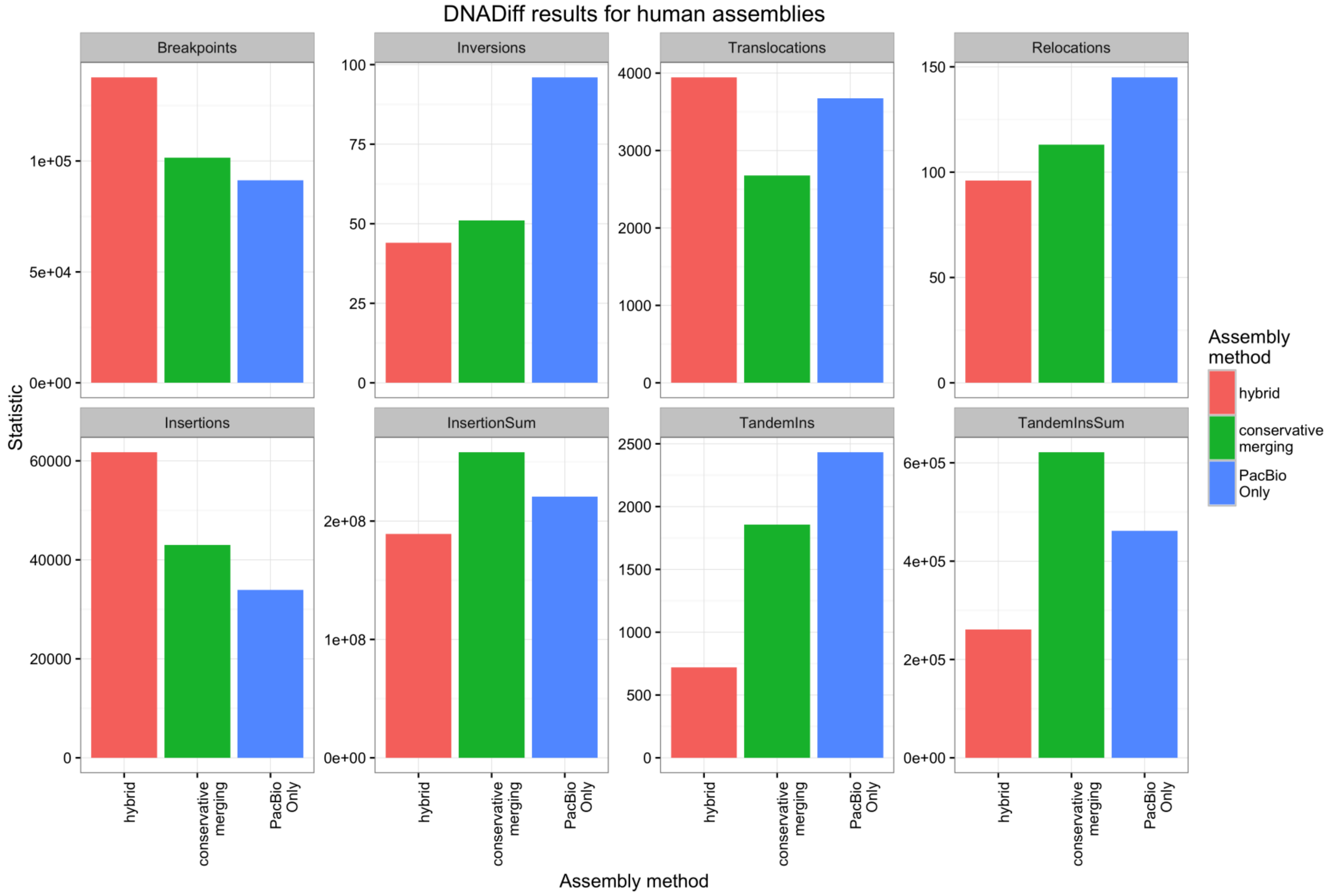

Supplementary Figure 12. Dnadiff results for hybrid, PacBio, and merged human assemblies. The conservative merged assembly is from merging the hybrid and the PacBio assembly using the parameters: hco=15,c=5.0,l=5000000.

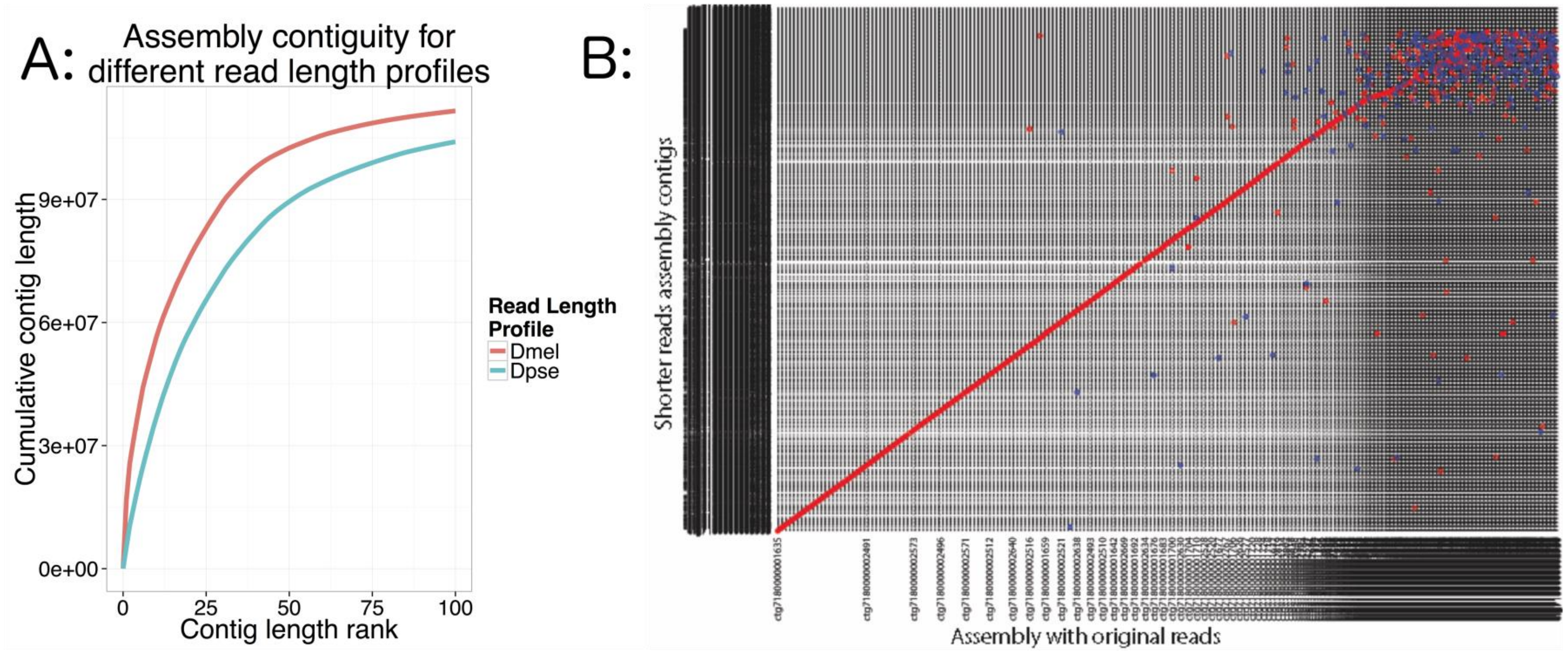

Supplementary Figure 13. A) Cumulative contig length distribution of a PacBio only assembly produced using the ISO1 long reads[2] and a PacBio only assembly using the same reads downsampled to resemble the length of the shorter D. pseudoobscura data. B) A mummer alignment dot plot illustrating the difference in contiguity between PacBio only assemblies produced using the same assemblies as in A.

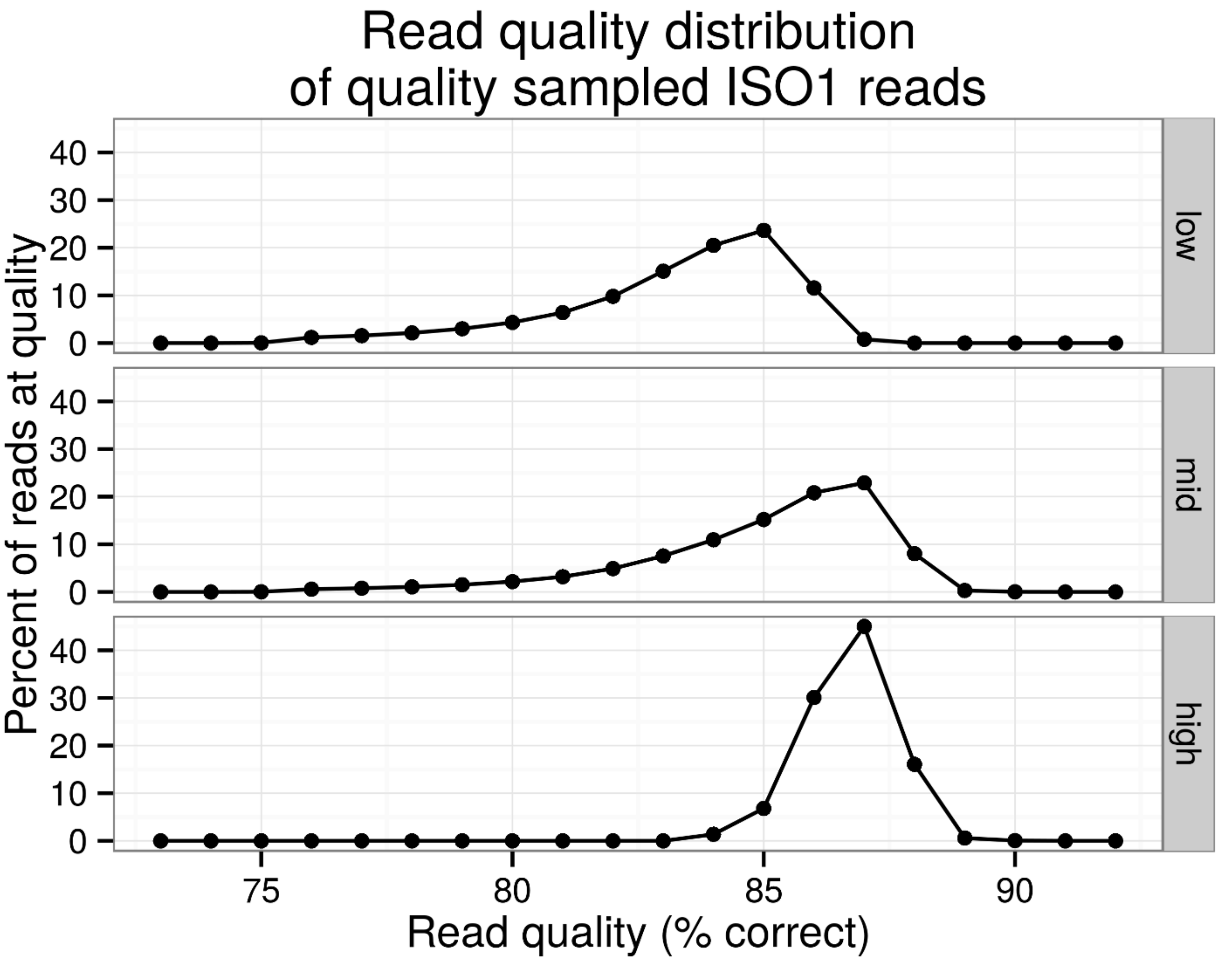

Supplementary Figure 14. Read quality distributions of the ISO1 dataset after downsampling to produce low (50% lowest quality reads), medium (a random 50% of reads), and high (50% highest quality reads) quality distributions.

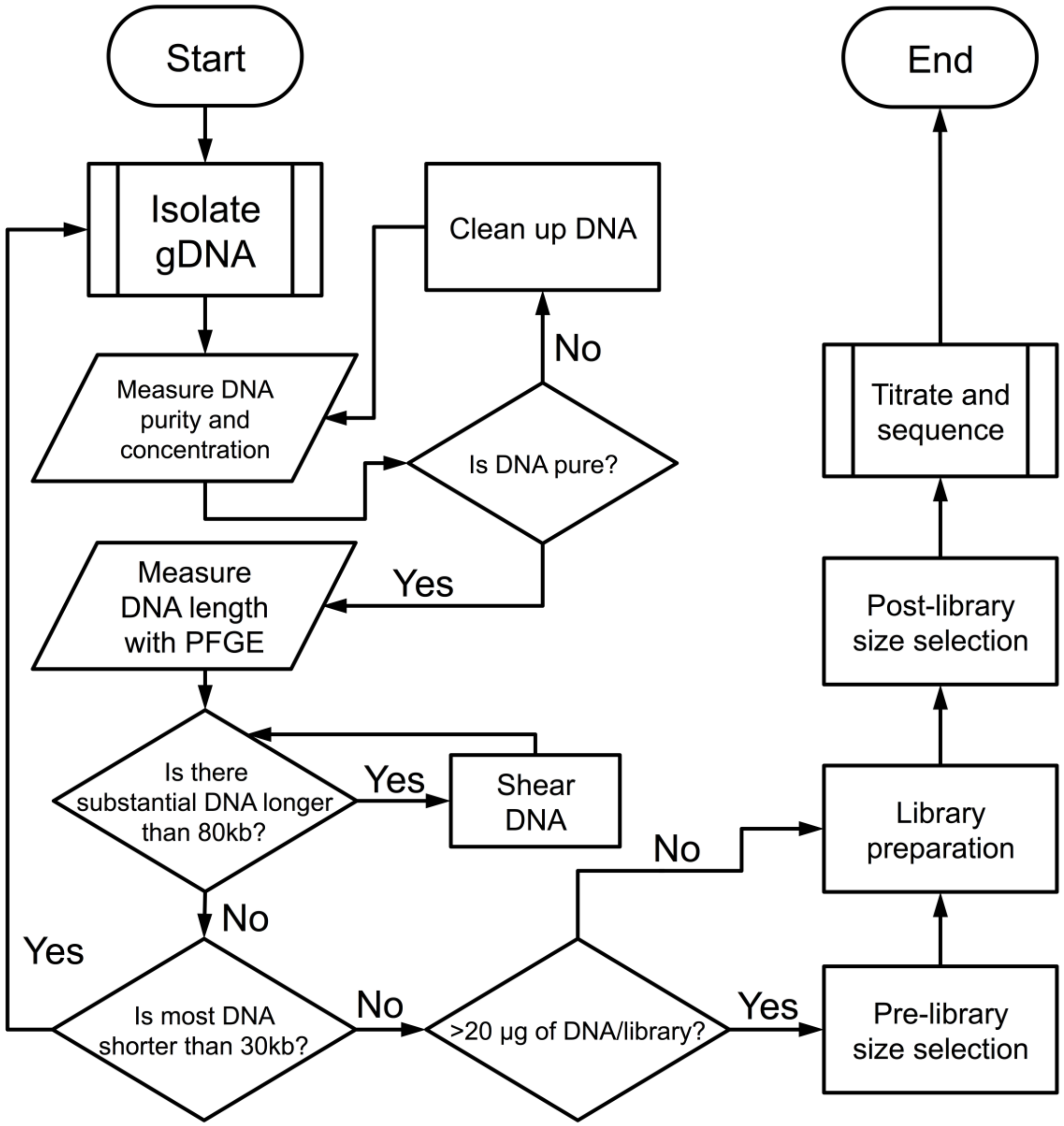

Supplementary Figure 15. Flow chart showing the key steps involved in in generating long reads for optimal genome assembly.

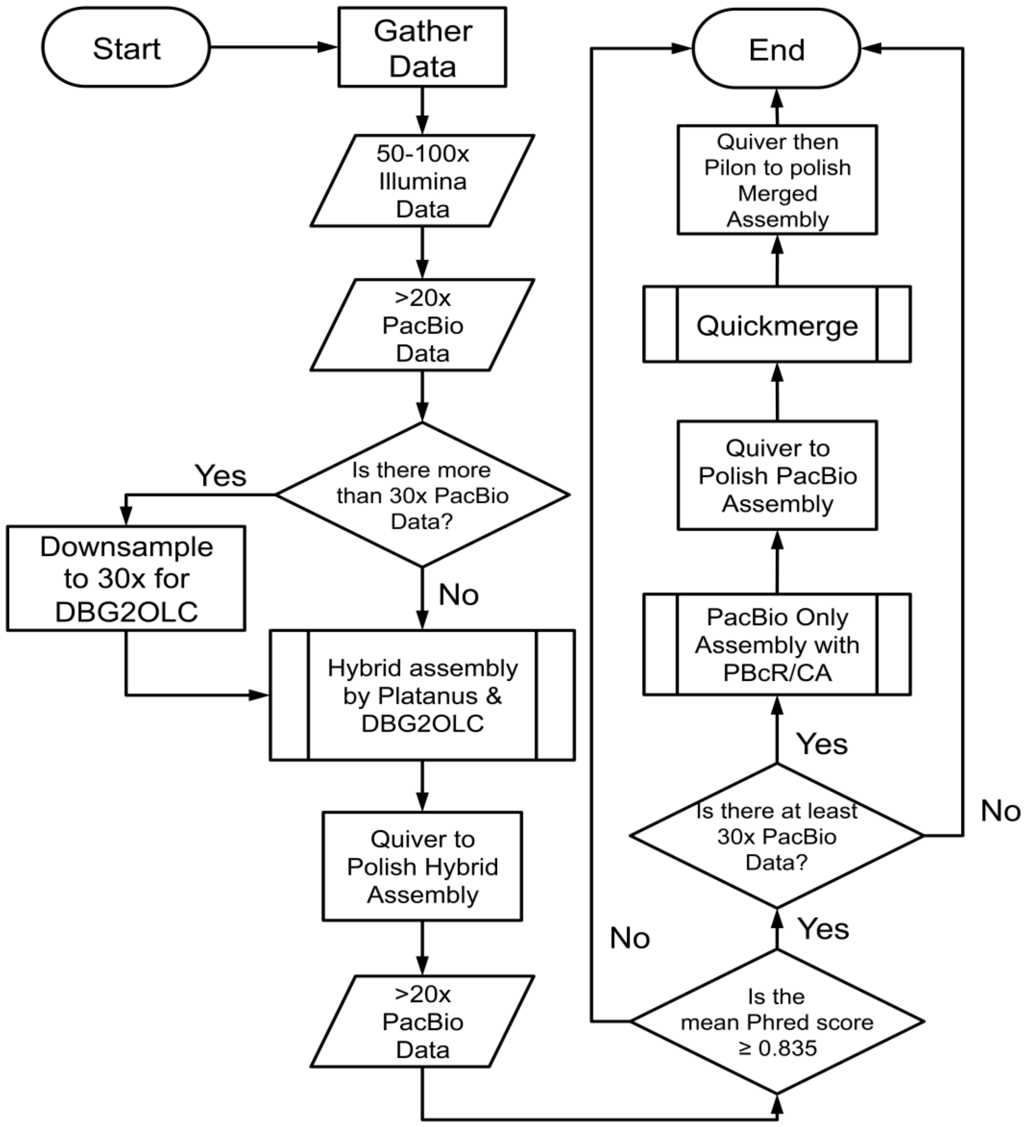

Supplementary Figure 16. Flow chart showing the key steps involved in assembling the long reads into a highly contiguous reference grade assembly.

Outline of the quickmerge algorithm:

1) Read delta file and store the start and ends of MUMs. Convert the delta format into tsv format and write it as a file called "aln_summary.tsv".

2) Check whether the queries are completely inside the reference sequences.

3) Check whether the query alignment is at the 5' or 3' end of the reference.

4) Do the same as 3 but check if the alignment is at the 5' or 3' end of the query.

5) Check if the query is on the forward or reverse strand (the reference is always on forward strand).

6) Calculate the total length of alignment for a reference and query pair.

7) Calculate the length of overlapping but non-aligning sequence in a reference-query alignment.

8) Calculate the length of sequence overhang – both for reference as well as query in an alignment.

9) Find the anchor/seed alignments based on the length and HCO cut-off.

10) Find the path through the alignment graph that represent the longest contiguous path through the HCO nodes. Reference contigs meeting the HCO and length threshold are used as initiation points for paths and extended in both 5' and 3' directions if an adjacent HCO node is available.

11) Generate the sequence corresponding to the path found in step 10, using contig sequences from reference and query assemblies.

Program commands and settings:

Downsampling

We used three different downsampling schemes on the *D. melanogaster* data: first, we randomly downsampled the data by drawing a random set of SMRTcells of data from the entire set of 42 SMRTcells; second, from those datasets, we downsampled the longest 50% and 75% of the reads. Finally, we downsampled the *D. melanogaster* data to match the read length

distributions of PacBio reads from a pilot *Drosophila pseudoobscura* genome project that was produced using a standard protocol without aggressive size selection (generously made available by Stephen Richards). We used the *lowess* function in R with a smoother span (f) of 1/5 to generate curves representing the distribution of read lengths in the *D. melanogaster* and *D. pseudoobscura* datasets, then assigned a probability to each read length defined as the quotient of the *melanogaster* distribution and the *pseudoobscura* distribution at that read length. Reads were then randomly removed from the *D. melanogaster* dataset according to the assigned probabilities. This method was used for all coverage up to 53X. Thus, we generated a read set that relatively closely resembles the read length distribution of the original *D. pseudoobscura* data, but is made up of *D. melanogaster* sequence data, allowing for a comparison of assembly quality with regard to read length without differences in the genomes of the two species as a confounding factor. The lowess function resulted in a slightly over-smoothed distribution such that samples drawn from it were slightly longer than in *D. pseudoobscura*. Consequently, assemblies from these reads should be slightly better than if they exhibited the (shorter) distribution for *D. pseudoobscura*. As such, this choice is conservative and, if anything, underestimates the importance of size selection.

Additionally, we downsampled based on read quality to test the effect of read quality on assembly contiguity. We used a custom script to separate the entire 42 SMRTcell ISO1 dataset into two halves. One half contained the 50% of all reads with the lowest average base quality, while the other half contained the 50% of all reads with the highest average base quality. Cutoffs were chosen for individual 100bp length bins, so the resulting datasets maintained the length distribution of the original data. We also generated a dataset containing 50% of the data that consisted of randomly chosen reads (to preserve the quality distribution of the original data).

PacBio only assembly

The spec file for all PacBio only assembly was as follows -

```
useGrid=1
scriptOnGrid=1
ovlCorrOnGrid=1
frgCorrOnGrid=1
```

```
ovlMemory=128
ovlStoreMemory=128000
threads=32
ovlConcurrency=1
cnsConcurrency=32
merylThreads=32
merylMemory=128000
frgCorrThreads = 16
frgCorrBatchSize = 100000
ovlCorrBatchSize = 100000
gridOptionsScript = -pe openmp 1
gridOptionsConsensus = -pe openmp 32
gridOptionsOverlap = -pe openmp 32
gridOptionsCorrection = -pe openmp 16
gridOptionsFragmentCorrection = -pe openmp 16
gridOptionsOverlapCorrection = -pe openmp 1
```

The pipeline was run as

```
PBcR -l dmel -s pacbio.spec -noclean -fastq dmel_all.fq genomeSize=130000000
```

Hybrid assembly

Platanus run options:

```
platanus assemble -t 64 -f /path/to/illumina/data.fa -o plat_assembly -m 512 1> plat_assembly_out.txt 2> plat_assembly_log.txt
```

DBG2OLC run options:

```
./DBG2OLC_Linux k 17 KmerCovTh 2 MinOverlap 20 AdaptiveTh 0.002 LD1 0 MinLen 200 Contigs illumina_contigs.fa RemoveChimera 1 f pacbio_reads.fa
```

```
sh ./split_and_run_pbdagcon.sh ../backbone_raw.fasta  ../DBG2OLC_Consensus_info.txt ctg_pb.fasta ./consensus_dir >consensus_log.txt
```

Assembly merging

Assembly merging involved the following steps:

1. nucmer is run to align hybrid assembly to PacBio only assembly.

nucmer –mumref –l 100 PacBio_only.fasta hybrid.fasta

2. Alignments are then filtered to retain unique alignments

delta-filter -i 95 –r –q out.delta > out.rq.delta

3. Then quickmerge is run.

quickmerge –d out.rq.delta –q hybrid.fasta –r PacBio_only.fasta –hco 5 –c 1.5 –l 1000000

At the end of the run four files are generated:

aln_summary.tsv: this file has all the mummer alignment output parsed in a table format.

summaryOut.txt: this file has all the stats computed by *quickmerge*.

anchor_summary.txt: this file contains information about all the anchor/seed alignments.

merged.fasta: this file contains the merged contigs. A quick quality check for merging is performed by aligning merged contigs onto PacBio only and hybrid contigs using nucmer (-mumref).

Spilcing joints:

>lcl|ctg7180000001635:1691416-1691616
TTAGTATTAAAGCAAATTAATCCAGCATGTCCGGAAATATGATGGAAATGTAAATCTTTCAATTTTTG
ATGGTTCTCTTTCATAAATTTAATTTCATTATAATTTTATTGATTCCTTATAAACATAATAATTTTCTTT
AAGAACTTCTGTCATGTTCTCAATTCTTATTTGCATACTTCTAAAATTGGAGTTAACATCTTC
>lcl|ctg7180000001635:4691869-4692069
TGGCTCATGGTGTGCAAATTGAGCGCTCATTTTTTATGGACCCAAATTATGCGAGGGGCGCTGCTC
TCTTCGCATATGCTTATTTATGAGATCTAATAATCGCTACATGCTGCCACGCCCCCTCACTTACACT
TGTACTTTCACCTCCCCCCTATTTTTCACCTTACAGTCTGGACTCGCGGGGCGGAAACGTGAAGTT
GG
>lcl|ctg7180000001635:5378403-5378603
CTAGGTCAGCCGAATACCTTTAAAATCCTTACTGAGTCTCTAATATGCGGAGTGAGTAGCTTCCCT
TCAGATGTCCTTAGTCCGTGTAGCTCCTGTCTCACACATTGCAAGTATTTGCTTTCCTTAAGCTTGT
CCTAGATAATCAGGAGTTCCTGAAATGTCAACGCTCATCTAGTATGCATAGTTTCATACAGCTAAGC
C
>lcl|ctg7180000001635:22183841-22184041
TTAACCACAGCCAGTGAGAAATATAAATACGAGCCGAAAGCACCCCAAACCAGAGCCAGATCAAAT
AATAATAACATTCAGACTGTCCTGCCAGGCAAACGGGTAACTTAATTACGGAAGCCACAGATTGTT
GCAGCAGTATGTGGTGTCGCCACAAACATTGCACTACCTTTTTGGGGTTTTCACTCAACGAGACGT
GTG
>lcl|ctg7180000001635:23794771-23794971
AAACAGAACTTGGACAAACAATCGATTTGAATCCCTAAACGGGATTAAAGTCGACCGCAAAGCGAA
GCGAAATTTGCAAAAATCCCTTGCGATTTGTCAGCCCAAATGGCAGAGCGGGTCTTATGAGTTTAT
CTAATCTGCATCGGTTGCAAGTGGGTTCGATCTACCTGATCACGGCTAGGGAAATCCTTACAAATC
CAC
>lcl|ctg7180000001635:25742895-25743095
TTAATTTATTATTTATTATTTATTATTTATTATTTATTATTTATTATTTATTATTTATTATTTATTATTTATT
ATTTATTATTTATTATTTATTATTTATTATTATTTATTATTATATTATTATTATTTATTATTTATTTATTAT
TATTTTTATTATTATTATTTATTATTTATTATTTATTAATTTATTATTTATTATTT
>lcl|ctg7180000001635:25750288-25750488
TTTATTATTTATTATTTATTATTTATTATTTATTATTTATTATTTATTATTTATTATTTATTATTTATTATTT
TATTATTTATTATTTATTATTTATTATTTATTATTTATTATTTATTATTTATCATTTATTATTTATTATTTA
TTATTTATTATTTATTATTTATAACGAATCAAATGTTTCAAGTGGCACATTAAGGT
>lcl|ctg7180000001635:27711702-27711902
AAAAAATATAAATAAGCGCGTTGTGTTATCAACGCTAGCAAAAATATTTGATCCGTTAGGATGGTTG
GCACAGTCACGGTTTCAGGAAAACTTTTTATTCAAAAACTTTGGATAAATAAAAGTGAATGGGATCA
GGAATTATCCATAGAAGATAAAAAATTATTGGGAAAAATATAAAGAAAATTTATTATTGTTAGAGAA
>lcl|ctg7180000001683:4358259-4358459
AACCTTTCACGTCCCCTGATGCGTGCCATCGGTGTGCTGGGGTACATATATCCTACGCCGATCCA
GGCCTCTACGATTCCTGTGGCTTTGCTAGGTCGCGATATCTGCGGCTGTGCTGCCACGGGAACG
GGCAAAACTGCTGCTTATATGTTGCCTACTCTAGAACGTTTGCTGTATCGTCCTCTTAACAACAAG
GCCATC
>lcl|ctg7180000001683:6043595-6043795
AATCTCATGCAATTACACGAAAACGTTGTTGACACGGCATACGAAAACCCAACCAAAACTCAGCCA
AAACCCACCCGAAGCCCGATTGTGTTTGCCCATCGGAATGGGCGATTATGTAATAATAACATGATA
GCCAACTAACTACACACTAATAGTACTGGAATGCCCCAGTCACTTGAACGCTTTGCTTTTGTTCAC
CAA
>lcl|ctg7180000001683:7128297-7128497

TTATTCAATAAGGTATCGTATATGTCTTACATTCTAACTAAGGCTTGATCCTTTGCTGGAGGATTATT
CCTTTGTAATACAATAAAATGTAGAATATATAGATACTAACATTTTTTAACATCTTACCGACGTCTTA
GAAGGGTATCGAAAAGCCCGTTGGAAAAACAATGAAAACACAATTTGATGTCTAAGCTTGACTCC
>lcl|ctg7180000001683:10285140-10285340
GCTCGAGTTTGGGAATTGTCTTCCTATTTTTTATAGGGTTGACTCTACTTTTGCTAGCTATTATATTA
ACATGAGGTCCTACTTTAGCATAGACTACTGCAGCATATGCTTTTTCGGAGGCGTCCGCAAATCCG
TGAATCTGAATGACTGAAGAACTGTTTGAATTAATCCACCTTGGGATTCGAATATTCTCTAACAATA
>lcl|ctg7180000001683:12810560-12810760
AGCATTCGATTGTGAGCATTCATTGGTAGATTTGTATTTATTTCTGTTCTTTTTTTTTTAAATTGTTTT
CATCGTTTGTACATTTTTTGTTTGGGATCCGGTATTTCTATGTGACAAGTCCGCATGTAAAGTATAA
TGTAAATTTTATAGTCAGCCGTTCTTTTGTTTTTCATTTTATCTTGGTACAATACTTTGTAAAAT
>lcl|ctg7180000001683:15841256-15841456
TGAATGGAAAACCGAATTATAAACTTCAGTGGCCATTAATTCAAAATTAGTCTCACCGTTTTGTATA
AAATATTGTGGCTTCATGTATGTTCAGGCATCAATGCCGAGGAAATCAATAGGGAGATAATAACTAT
GATAACTAATCCTTAAATGGTATGTTTAATAACTTTTTACACTGCAAAGACAATAAAAAGGATAAAA
>lcl|ctg7180000002491:18609-18809
TTTAGATTGCCTCTCATTTTCTCTCCCATATTATAGGGGAAATATGATCGCGTATGCGAGAGTAGTG
CCAACATATTGTGTCTTTGATTTTTTGGCAACCCTTTTTTGGCAACCCAAAATGGTGGCGGATGAAC
GAGATGATAATATATTCAAGTTGCCGCTAATCAGAAATAAATTCATTGCAACGTTAAATACAGCACA
>lcl|ctg7180000002491:1721776-1721976
AGGATGAGGAGAAACGAGGAGATGCCCCAGAGGATGCCCCCCAAAGAAGCTGCTTAGATTTAGG
CTAAGAATCGATGAAACGACGGAGTGAAAGATAGACGGGTGTAGTTGTGCATATATCGAGGGTAA
CTGTACCATAAGTACACAGCAACACTTAGTTGCATTGCATAAATAAATGTCTCAAGTGAGCGTGATA
TAAGA
>lcl|ctg7180000002491:6176867-6177067
TTACAGCCTACTCTATTTTAAAGTTGCCTTTAATACAAAAATGACTTGTATCTGAATTCCTAATCTTC
ACATCCTCTTTTGCTATTACCCATTTTCCAGAAATAAACTAAAGCTTCAACAACAATTAATTAAGTAT
TCATACGGCTCACTAAAATACGTAGTACGTTTTAAACTATCGCAACATTGATAAAATGTTAAGAT
>lcl|ctg7180000002491:6368663-6368863
GTGCGTCGGACAATTCGTTCATCAAGAAGCTCAAGGCATCCGGACGCAGTCTGGTCGTCTTCTAT
GGCTCACAGACCGGAACCGGCGAGGAGTTCGCCGGCGTCTGGCCAAGGAGGCATTCGTTACCGT
CTGAAGGGCATGGTTGCCGATCCGAGGAATGCGACATGAGGAGCTGCTGCAGCTGAAGGACATC
GACAACTC
>lcl|ctg7180000002491:15142863-15143063
ACAAAACACCCCGAAACAAATTTTTATATTTATAGTGTAGACAGAATTTTAATTTTTTTTATTTCATTA
GACCTTCAACTTTTAAAAAATGAAATACAAATATTCAAAAATATATGCAATTCCCAATTAGAATTTAA
GAATGAAATGATTTAGATTTGCCAGATTATATTTTATTTCAGTTTGCTGCTGTTTAGTTATAAA
>lcl|ctg7180000002491:20203285-20203485
CACTCGGTGACCGAAAAGGTGTCTTCCTCACACTCCGCTAAGGTGTCTTCATCGAGTCGCAGGAT
TGTCTGATAGTGTGATACTGGGTATACGAGGACCTTTTTATTTTTCACTGGGATACTTGTTTGGATT
GTAAAAAGTCTTTAACAACTGAAAAGCAGTCTAATCTAGCGAGTGTTTCGGACAAAATTCTTTTCAT
CT
>lcl|ctg7180000002491:21423134-21423334
GGTTTCTGCTACCGCCAAGAAGCCGAAAGCGAAGACTACGGCTGCCAAAAAGTAAATTGTGAAAA
AGTGCAGTATTTGGTACATGTTCGCAATTAAAATTTAGATTTATGATTTATAGATCTGAAATTTGTTT
AAACAAGTCTTTTCAGGGCTACAACGTTCCGTTGCAAGAGAAAAAAATTTTATTTTCTTCCACTTATT
>lcl|ctg7180000002496:6132599-6132799
CATGTCGCCTTAGATAACAAAATCCTAAATATAATTTATCGCTCTCGATTCATTTACATAAGATATGA
ACGGAGCCCAAAATTGTAAGTCTTTAAATATATTCGTGTTCATGTGTGAACAGCAATGTTGTTGTTT
TGTGTTAAGTTGTCAAGTTTCTAAGCGCATACCTAAAGATATAGCACCAAGATATTCTATAGACAA
>lcl|ctg7180000002496:10848188-10848388

AAATCCTAAATATAATTTATCGCTCTCGATTCATTTACATAAGATATGAACGGAGCCCAAAATTGTAA
GTCTTTAAATATATTCGTGTTCATGTGTGAACAACTATTGCTCATCTTTTAGGAGTAATAGACAAACC
ACTTAAATATCTTATGTTCCGAAGAATAAGGCCAATACTATTATATTTAAATTTAAATAACCTCA
>lcl|ctg7180000002496:13783777-13783977
CATTTAATGAGTTGGTCGCTTTGACTGTTTATCGTTTTATTAGTTGAGCAGTTGCACCATGTTTAAA
CTGCCAAATCAAATGTTACACTGAAATTATGCTTCTTATCTACTGTACTGTTCCACTGACTTTTTCTT
CCGTCATTGACTTAAGTTAGTTTACATTTATTATTTTTGCGTTTTTTATTACATTGCAATCAATAA
>lcl|ctg7180000002496:15132313-15132513
GTTTGATCATTCGAATAGTGCGATCGGAGGCCATTCGACGAAGCCATGCTCCATCAAAACGCCAA
ATCCTCCGAAGACAGCAATTCTGGCTGCTTGCCGTTCATACCTGCAGGAGAGAGCTCCCCAAATT
GCGCTCTTCGCTGCCCACTGCCCACTGCGCTGCGGCTGCGACGCCTGCATCGCTGGTGGCTGCT
CTCGAAA
>lcl|ctg7180000002496:16017977-16018177
ATATATACATATATACATATATACATATATACATATATACATATATACATATATAATATATACATATATA
CATATATACATATATACATATATACATATATACATATATACATATATACATATATACATATATACATATA
TACATATATACATATATACCATATATACATATATACATATATACATATATACATATATACA
>lcl|ctg7180000002510:27592-27892
AAATTTGAACTCGAACAAAAAAGTACACTTCTACCCTTTAAACGAAATTACGATAATAAAGATACAAA
AAATAAATATATCTGAAAAAAAAATCAGGTCTGATTTCTACAATTGTTAAATATGATAACAAGTCCCA
ACAATCCTTTCAGTATAGAACAAAACATGGAATATACTTAAGCCATGTGAAAGAACACATAACATAT
TAATATTCTAAACAATTTGAGCAGTTTTCTTGAGTTT
ACGCCTCCAACAATTTATCCTTCACCTTTCTTGAGTTCCAAATTCCTGGACCGTGATCAAG
>lcl|ctg7180000002767:1263476-1263676
ATCACAAAATAAATTTCAAAAATGACTTTAATTAGAATATTTGTCATAGAGGATTCAGTTGCGGCGT
GTGAAAAATAAAAAACTAAGGCAATGATTGTTGATTGTGTCCGCACTTCGTGCCTCAAGATATGAC
AAAAACAAAGACACCAGAATAATTCTAGTGTCTTTGATGTGACTTGTTGTTGCGATAAATGCAGATC
G
>lcl|ctg7180000002767:1577359-1577559
CAAGATATTCAACACTCGTCTTTAGGAATGAGCACTTGTTCATGTTAAACGAAAATACCGCACTAGA
TAACCTGTTCAACACGTCCCTCAGTCTAACAAAGGCCTCCCTCCTTTGTGCCAGCTACGACTAAAA
CATCATCTATGTAAACAACAGCATATCAGTATGCTACCTCACCTAATGCTTTTATTATGGATCGCTG
A

References:

1. Berlin, K. et al. Assembling large genomes with single-molecule sequencing and locality-sensitive hashing. *Nature biotechnology* **33**, 623-630 (2015).

2. Kim, K.E. et al. Long-read, whole-genome shotgun sequence data for five model organisms. *Scientific data* **1**, 140045 (2014).
